# Left diaphragmatic hernia after a mild blunt trauma in Syria: a case report

**DOI:** 10.1093/jscr/rjad100

**Published:** 2023-03-21

**Authors:** Jameel Soqia, Jamal Ataya, Salem Algomaa Alhadid, Ameer Kakaje, Hussain Chaban

**Affiliations:** Faculty of Medicine, Damascus University, Damascus, Syria; Faculty of Medicine, University of Aleppo, Aleppo, Syria; Faculty of Medicine, Damascus University, Damascus, Syria; Faculty of Medicine, Damascus University, Damascus, Syria; Faculty of Medicine, Damascus University, Damascus, Syria

**Keywords:** diaphragmatic hernia, late-presenting hernia, left sided hernia, upside-down stomach, trauma

## Abstract

Left side traumatic diaphragmatic hernias (DH) are very rare and usually present acutely. They might represent after years of minor trauma, and they should be considered among differentials to avoid complications. We present a 28-year-old female coming with acute epigastric pain radiating into the chest with dyspnea and vomiting. Her history was negative for trauma and other than very minor trauma two years earlier. Chest X-ray showed atelectasis with mild pleural effusion. Computed tomography scan showed several cavities, filling the left chest with a gaseous liquid level. Surgery was performed that demonstrated DH and the abdominal viscera were returned to the abdomen without any complications. Traumatic DHs can be easily overlooked with the absent of recent major trauma. They can represent years after the original trauma with acute symptoms, which can make it hard to diagnosis if not considered.

## INTRODUCTION

Traumatic injury to the diaphragm is relatively uncommon, with an incidence between 0.8 and 8% [1]. We present a young female who had acute abdominal pain and dyspnea. No significant history of trauma was found that highlights the importance of keeping diaphragmatic hernia (DH) among differential diagnosis of unexplained acute abdominal pain.

## CASE PRESENTATION

A 28-year-old housewife who presented with dyspnea and vomiting for one week, with epigastric pain radiating into the chest. She had no fever, cough or hemoptysis. She had no significant history for cardiac or pulmonary disease, and she was a non-smoker. She did not take any medication, including oral contraceptive pills. No significant family history other than diabetes and high blood pressure was found. The patient recalled carrying heavy weights around a year ago without a previous trauma story, and she had a subsequent dyspnea with a compressive intermittent chest pain that increased after eating at that stage, which all resolved spontaneously after a few days. She had a history of three Caesarean sections, with the last operation being four years ago.

On examination, vitals were within normal range. However, high-pitched sounds were noticed in the left hemithorax. Cardiovascular and abdominal examination were otherwise remarkable. Her BMI was 23.4 kg/m^2^. Other systems were also normal.

**Figure 1:**
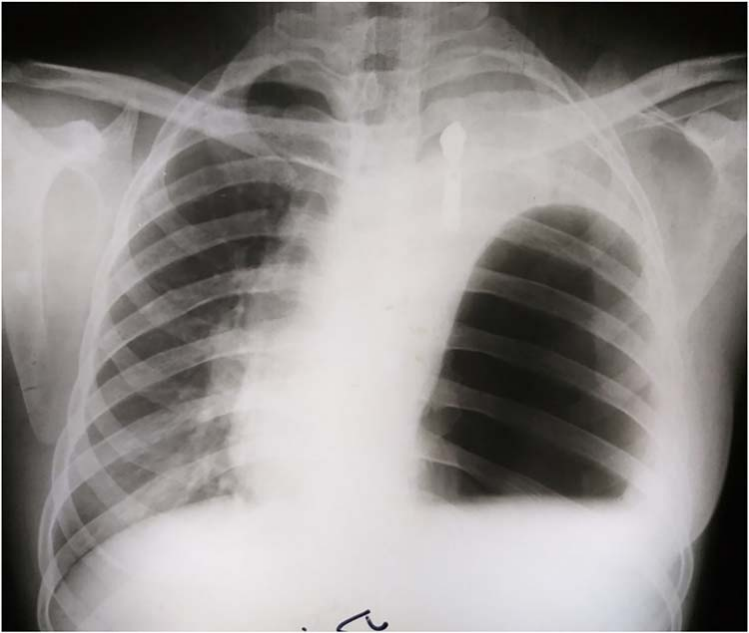
Presurgical CXR.

**Figure 2:**
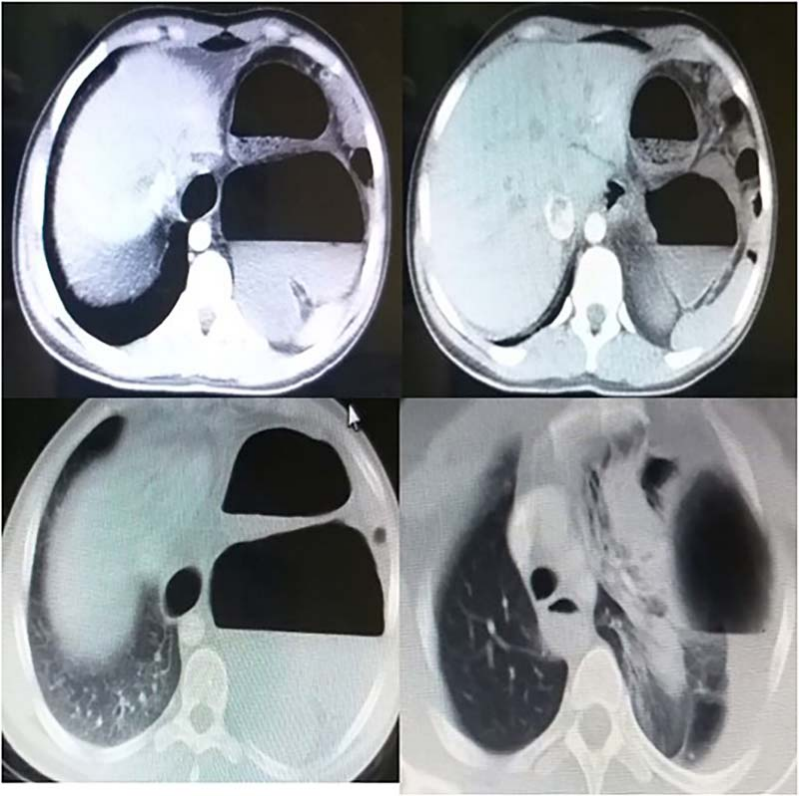
Presurgical CT scan.

Laboratory tests showed white blood cells count of 9000/microliter with neutrophils 82%, mild anemia (Hb = 10 g/dl) and respiratory alkalosis (pH = 7.46, pCO2 = 28, and HCO_3_ = 20). Chest x-ray (CXR) of the chest was not able to visualize the left hemidiaphragm with a hollow viscus in the left thoracic cavity and a right shift of the mediastinum (Fig. 1). Computed tomography (CT) scan showed several viscera in the left thoracic cavity, containing air-fluid levels with the fluid not being homogeneous, pushing the left lung, the heart and the mediastinum to the right (Fig. 2). The CT also showed dilation of the esophagus with a presence of a fluid-gas level (Fig. 2). Additionally, there was atelectasis in the left lung. Finally, the liver and pancreas were in their normal position. An endoscopy was performed, which visualized the DH, which was 3 cm.

**Figure 3:**
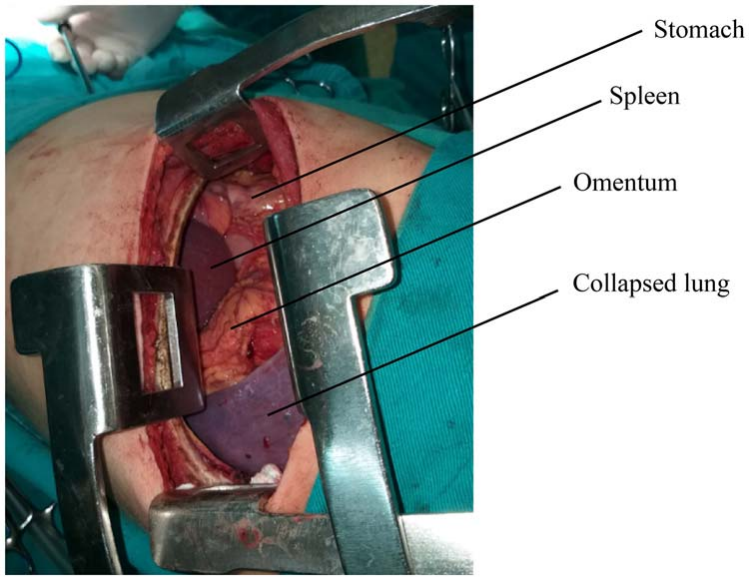
Abdominal viscera during the surgery.

The surgical procedure was performed under general anesthesia with double-lumen tube and high airway pressure with low ventilation. Left lung was isolated, and a left lateral thoracotomy through the seventh intercostal space was performed. The spleen, stomach, omentum and part of the colon were found in the left chest, as shown in Fig. 3.

**Figure 4:**
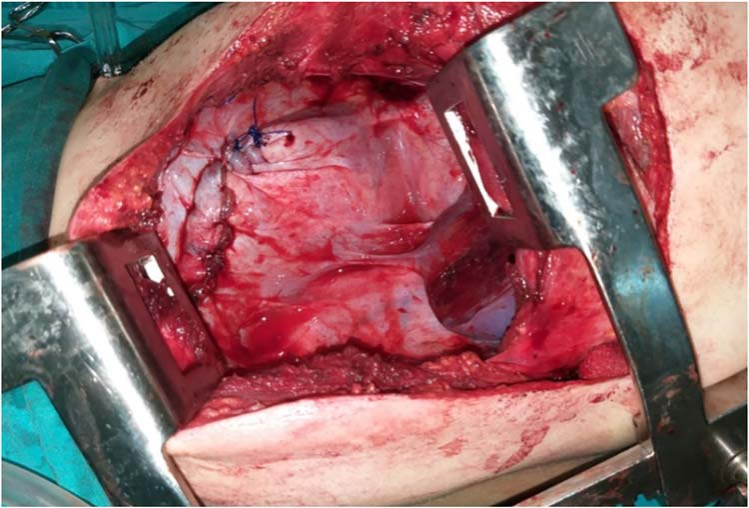
Diaphragm after repair.

The diaphragm, which was found torn in its muscular section, was reached, and the abdominal viscera was returned to the abdomen. The diaphragm was then repaired, and the left lung was well disseminated (Fig. 4).

**Figure 5:**
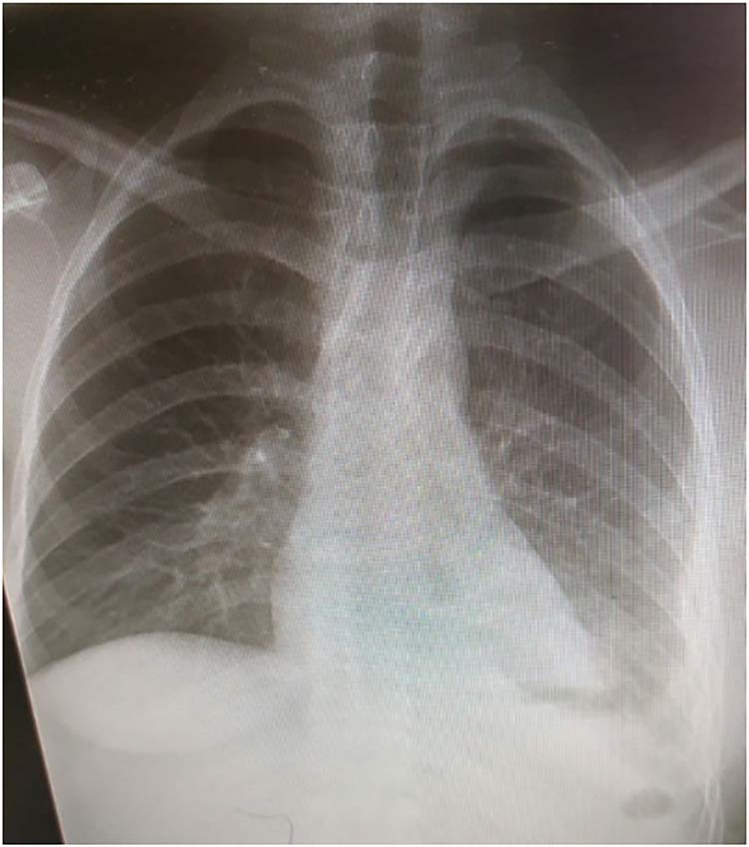
CXR postsurgery.

The patient was followed up with series of CXRs and patient is back to baseline (Fig. 5).

## DISCUSSION

The exact incidence of DHs is difficult to determine, given that many cases are asymptomatic and go undiagnosed. Furthermore, the incidence of blunt versus penetrating trauma is widely variable [1–3]. Males between 30 and 45 years have the highest prevalence of traumatic DHs [1–3]. Left DHs are more common compared to right by (60–70%) [2, 3]. This might be explained by the left side being relatively weaker and the liver playing a protective role in the right side [1–3] and therefore wounds on the right side require greater force to cause injury [3].

Most diaphragm injuries are associated with severe complications such as hemothorax, pneumonia, sepsis, injuries to the chest and abdominal organs, and a high-mortality rate can be up to (20%) [1–3]. The classification of traumatic hiatal hernias is based on two factors: etiology and time of onset, which have an important influence on the signs and symptoms of the injury [4].

Diaphragm defects are not easily detected because there are often no direct signs and symptoms. Moreover, most accompanying symptoms are nonspecific [5].

High clinical suspicion is required to make the diagnosis of diaphragmatic rupture; CXR is usually the first hint regarding the possibility of a diaphragm injury [4, 6]. In the case of exploratory laparotomy, careful examination of both diaphragms should be performed [7].

In this case, when the woman was presented, there was a large hernia with much protruding viscera, which led to a deterioration in the general condition, and thus a significant increase in the risk due to the size of the hernia being 3 cm. Therefore, the patient was rapidly managed due to the concerns of developing incarcerated hernia where mortality rates are very high [8]. Such procedures can also be carried out endoscopically [9], but in our case, the chronic nature of the hernia made open surgery extremely challenging due to the difficulty with returning the abdominal components.

CXR might give a misleading diagnosis as it might resemble a hydatid cyst or pneumothorax, which might lead to mismanagement such as inserting a chest tube, which might cause a perforation in the stomach and colon; therefore, great care should be taken when dealing with other similar cases [10].

## CONCLUSION

Traumatic diaphragmatic hernias are rare and can be caused by relatively minor trauma that might be direct or indirect. This can occur even years after the initial trauma. Early diagnosis and management of symptomatic causes is crucial to avoid serious complications.

## Data Availability

Data will be made available upon reasonable request.
